# Circulating GLP-1 Levels as a Potential Indicator of Metabolic Syndrome Risk in Adult Women

**DOI:** 10.3390/nu13030865

**Published:** 2021-03-06

**Authors:** Min Joo Seon, So Yoon Hwang, Yujeong Son, Juhyun Song, Oh Yoen Kim

**Affiliations:** 1 Department of Health Sciences, Dong-A University, Busan 49315, Korea; fulme2@naver.com (M.J.S.); soyoon6766@naver.com (S.Y.H.); 2 Department of Food Science and Nutrition, Dong-A University, Busan 49315, Korea; ugson95@naver.com; 3 Department of Anatomy, Chonnam National University Medical School, Hwasun 58128, Korea

**Keywords:** glucagon-like peptide-1, metabolic syndrome, early indicator, women

## Abstract

Glucagon-like peptide-1 (GLP-1), an incretin hormone, plays an important role in regulating glucose homeostasis. In this study, the applicability of circulating GLP-1 levels as an early indicator of metabolic syndrome (MetS) risk was examined. Women without diagnosed diseases were grouped according to their number of MetS risk factors (MetS RFs) (no RFs as Super-healthy, *n* = 61; one or two RFs as MetS risk carriers, *n* = 60; 3 ≤ RFs as MetS, *n* = 19). The circulating GLP-1 levels and homeostasis model assessment insulin resistance (HOMA-IR) scores were significantly higher in the MetS group than in the other two groups. The GLP-1 levels correlated positively with adiposity, HOMA-IR, blood pressure, and high sensitivity C-reactive protein (hs-CRP), but not with fasting glucose and lipid profiles, whose significances were maintained after adjustments for age, smoking and drinking habits, menopausal status, and total calorie intake. The GLP-1 levels also increased proportionally with the number of MetS RFs. In the MetS group, the GLP-1 levels were much higher in individuals with obesity (body mass index ≥ 25 kg/m^2^). In conclusion, the circulating GLP-1 level may be applicable as a potential early indicator of MetS risk in women without diagnosed diseases. Further study with a large population is needed to confirm the conclusion.

## 1. Introduction

The metabolic syndrome (MetS) is a complex of various metabolic disorders, such as a low concentration of high-density lipoprotein cholesterol (HDL-C), elevated levels of triglyceride (TG) and blood glucose, raised blood pressure (BP), and obesity, particularly, central obesity [1,2,3]. Any combination of such abnormalities can lead to the development of chronic degenerative diseases, such as type 2 diabetes (T2D), cardiovascular disease (CVD), stroke, vascular dementia, and fatty liver disease.

According to the 2020 National Health and Nutrition Survey report from the Korea Centers for Disease Control and Prevention [4], one in three adults (especially two in five men) was obese (i.e., body mass index (BMI) ≥ 25 kg/m^2^), one in 10 had diabetes, two in five had dyslipidemia, and one in three suffered from hypertension [4]. The global prevalence of overweight and obesity is expected to reach 57.8% by 2030 [5], with the incidence of T2D doubling from 2.2% in 2000 to 4.4% in 2030 [6].

Adiposity, particularly abnormal fat accumulation, is a key MetS-causing factor since it has a strong influence on insulin sensitivity and β-cell function and thereby increases the risks of CVD and T2D [7,8]. It was found that abdominal obesity accounted for approximately two-thirds of the strongest factors contributing to adipose tissue-derived insulin resistance (IR) in both men and women [9]. Since IR will lead to a gradual progression from hyperinsulinemia to glucose intolerance and eventually, T2D [10], the early detection of MetS risks (including IR) is important for the prevention of chronic metabolic diseases [9].

Recent lines of evidence have indicated that glucagon-like peptide-1 (GLP-1), an incretin hormone, plays an important role in regulating glucose homeostasis [11,12,13]. In several clinical trials, the unfavorable effects resulting from the loss of incretin in patients with obesity as well as prediabetes and T2D were ameliorated after treatment with incretins or an incretin agonist [11,12,13,14,15,16]. In addition, fasting levels of plasma GLP-1 were significantly higher in patients with T2D than people with normal glucose tolerance (NGT), but the postprandial plasma GLP-1 area under curve was significantly lower in patients with T2D than people with NGT [11,14]. Furthermore, previous studies reported sex differences in circulating fasting GLP-1 levels and the GLP-1 response to oral glucose tolerance (OGTT). According to the report by Færch K et al. [11], women had lower fasting GLP-1 levels than men, whereas women had higher GLP-1 levels at 30 and 120 min during OGTT, and total GLP-1 responses than men. In addition, people with prediabetes or T2D had a lower GLP-1 response than those with NGT, which was most pronounced in women. Moreover, Toft-Nielsen MB et al. reported that males had a smaller response than females [14]. When food is ingested, the proglucagon molecules in specific intestinal enterocytes (named L-cells) are cleaved to form amidated peptides that are 30 amino acids in length, named GLP-1(7-36) amide (NH_2_), the predominant secreted form of GLP-1 which are then secreted into the circulatory system [17]. Once GLP-1(7-36) NH_2_ reaches the pancreatic β-cells, it binds to the GLP-1 receptor (GLP-1R) on the β-cell surface to induce insulin secretion, aided by increased intracellular Ca^2+^ levels. This process is inactivated when the plasma glucose level falls below 5 mmol/L.

Additionally, GLP-1-mediated signaling reduces the expression of proinflammatory and proapoptotic factors, thereby improving the viability of pancreatic β-cells [18,19]. The active GLP-1 has a very short half-life of 1–2 min in circulation, since it is rapidly deactivated by dipeptidyl peptidase-4 (DPP-4) [20]. Thus, the abundance of active GLP-1 molecules released from the intestinal L-cells is largely reduced by the time these molecules reach the portal vein, and only a small fraction remains in arterial circulation [21]. When glucose levels are higher than normal fasting levels, GLP-1 will also repress glucagon secretion from the islet α-cells, which aids the activity of postprandial insulin in mediating the anabolism of consumed nutrients [17,20]. Additionally, the release of a small amount of GLP-1 from neurons in the nucleus tractus solitarii [22], which is regulated by food intake and meal composition [23,24,25,26,27,28,29], contributes to satiety by regulating energy consumption and reducing gastric motility [30,31,32]. For example, the consumption of an olive oil-enriched Mediterranean diet for 28 days by individuals with abdominal obesity and IR resulted in a significantly higher blood GLP-1 concentration [33]. These findings suggest that an adjustment of the dietary composition can serve as a promising lifestyle strategy for managing obesity and diabetes by promoting GLP-1 secretion and its subsequent effects [31].

Most research studies have focused on the treatment of diabetes using GLP-1 agonists [15] but there have not been many strategies developed to prevent chronic degenerative diseases. Therefore, this study was carried out to examine the potential use of circulating GLP-1 levels at a stable condition as an early indicator of MetS risk in individuals who have no diagnosed diseases.

## 2. Materials and Methods

### 2.1. Study Participants and Design

The study participants, who were all Korean women (age ≥ 19 years old), were recruited through a public advertisement. Individuals with any chronic disease (i.e., diabetes, CVD, stroke, cancer, coronary heart disease) or other metabolic diseases (i.e., thyroid, renal, vascular, and liver disorders) were excluded from the study as were those taking antihypertensive, lipid-lowering, antidiabetic or antithrombotic medications. Among the recruited women (*n* = 169), 140 were finally included in the analyses. On the basis of criteria set by the modified National Cholesterol Education Program-Adult Treatment Panel III and the Korean Society for the Study of Obesity, MetS was defined as a status of having three or more of any of the following risk factors (MetS RFs): Fasting blood glucose ≥ 100 mg/dL; waist circumference (WC) ≥ 85 cm (for females); systolic BP ≥ 130 mmHg or diastolic BP ≥ 85 mmHg; TG ≥ 150 mg/dL; and HDL-C < 50 mg/dL (for females) [1,34]. Using this definition, the subjects were divided into three groups according to the number of MetS RFs: Those who have zero MetS RFs as “Super-healthy” (*n* = 61); those who have one or two MetS RFs as “MetS risk carrier” (*n* = 60); those who have three or more MetS RFs, as “MetS” (*n* = 19). The participants were given an explanation about the aim of the study, and all of them provided a written informed consent to the use of their data. The study was approved by the Institutional Review Board of Dong-A University (project identification code: 2-104709-AB-N-01-201603-BR-001-10).

### 2.2. Basic Information and Diet Survey

The diet survey was conducted through face-to-face interviews and consisted of questions regarding regular dietary habits as well as a 3-day diet record using the 24-h recall method (2 week days and 1 weekend). A semi-quantitative food frequency questionnaire composed of 32 questions based on the Korean National Health and Nutrition Survey form was included. The calorie intake and nutrient contents from the 3-day diet record were estimated using the Computer Aided Nutritional analysis program (CAN-pro 4.0; Korean Nutrition Society, Seoul, Korea).

### 2.3. Anthropometric and Blood Pressure Measurements and Blood Collection

The height and weight of each participant wearing light clothing but no shoes were measured, and the BMI was calculated as the body weight divided by the height in square meters (kg/m^2^). The WC was measured after normal exhalation, with the person standing. Using an automatic BP monitor (HEM-7220; Omron Healthcare Co., Ltd., Matsusaka Factory, Japan), the systolic and diastolic BPs were measured from the arm (left or right) of the person in the seated position after 20 min of rest. Blood samples were collected in both plain and ethylenediaminetetraacetic acid (EDTA)-treated tubes in the morning after an overnight fast. The samples were centrifuged at 1300× *g* for 15 min for plasma collection and at 2000× *g* for 15 min for serum collection, both at ambient temperature, and then stored at −80 °C before analysis.

### 2.4. Lipid Profiles and Glycemic Parameters

Serum concentrations of TG, total cholesterol (TC), and low-density lipoprotein cholesterol (LDL-C) were measured with commercial kits on a Hitachi 7150 auto-analyzer (Hitachi Ltd., Tokyo, Japan). After precipitation of the serum chylomicrons with dextran sulfate magnesium, the concentration of HDL-C in the supernatant was measured enzymatically. The fasting serum glucose concentration was measured using the glucose oxidase method with a Beckman glucose analyzer (Beckman Instruments, Irvine, CA, USA). Insulin and C-peptide levels were measured by radioimmunoassay using commercial kits (Immuno Nucleo Corporation, Stillwater, MN, USA). The IR level was calculated by the homeostasis model assessment (HOMA) according to the following equation: HOMA-IR = (fasting insulin [µIU/mL] × fasting glucose [mmol/L])/22.5 [16]. The glycated hemoglobin (HbA1c) level was measured using the Variant II TURBO HbA1C Kit-2.0 (Bio-Rad, Hercules, CA, USA) on the Variant II TURBO testing system (Bio-Rad).

### 2.5. Serum High-Sensitivity C-Reactive Protein Determination

The serum level of high-sensitivity C-reactive protein (hs-CRP) was measured with an ADVIA 1650 system (Bayer Healthcare, Tarrytown, NY, USA) using the High-Sensitivity CRP-Latex (II) × 2 Kit (Seiken Laboratories Ltd., Tokyo, Japan).

### 2.6. Glucagon-Like Peptide-1 Determination

The plasma GLP-1 level was measured using a commercially available quantitative Total ELISA kit (EMD Millipore, Billerica, MA, USA), with the microplate absorbance reader (Bio-Rad) set to 450 nm. The assay was performed in duplicate.

### 2.7. Serum 17ß-Estradiol and Follicle-Stimulating Hormone Determination

Serum levels of 17ß-estradiol and follicle-stimulating hormone (FSH) were measured using commercially available kits (ADVIA Centaur Estradiol Assay and ADVIA Centaur FSH Assay, respectively; Siemens Healthineers, Cary, NC, USA) on the ADVIA Centaur immunoassay system (Siemens Healthineers).

### 2.8. Statistical Analysis

All statistical analyses were carried out using SPSS version 25.0 (SPSS Inc., Chicago, IL, USA). To evaluate differences among the groups, the one-way analysis of variance with the *Bonferroni* method and the general linear model with adjustment for confounding factors were used. Pearson and partial correlation analyses were used to determine the associations between the GLP-1 concentration and MetS RFs. The results are expressed as the means ± standard errors, percentages, or correlation coefficients. Differences with two-tailed *p*-values less than 0.05 were considered statistically significant.

## 3. Results

### 3.1. Baseline and Biochemical Characteristic According to MetS Status

Table 1 shows the baseline characteristics and biochemical parameters of the 140 participants according to MetS status: Super-healthy (*n* = 61), MetS risk carriers (*n* = 60), and MetS (*n* = 19). The MetS group were older than the other two groups. Compared with the individuals in the Super-healthy group, those in the other two groups were obese, especially the women in the MetS group, and had higher BP and WC measurements. Moreover, a significantly higher proportion of the women in the MetS risk carriers and the MetS groups were in menopause. There were no significant differences among the groups in terms of daily total calorie intake (TCI), proportions of calorie intake derived from macronutrients, and proportions of current smokers and alcohol drinkers. The levels of glycemic indicators (i.e., fasting glucose, HbA1c, and C-peptide) were the highest in the MetS group. Compared with the levels in the Super-healthy group, the fasting levels of TG, hs-CRP, and FSH were significantly high in the MetS risk carriers group and even higher in the MetS group. By contrast, the blood HDL-C and estradiol levels were significantly lower in the MetS risk carriers and MetS groups.

### 3.2. Circulating GLP-1 and HOMA-IR in the Study Participants According to MetS Status

**Figure 1:**
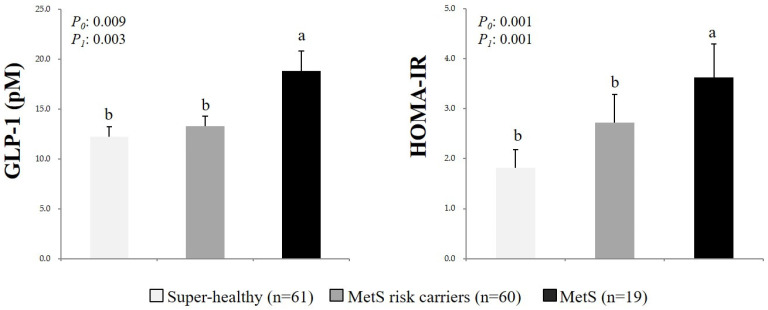
Circulating glucagon-like peptide-1 (GLP-1) levels and homeostasis model assessment insulin resistance (HOMA-IR) scores in the study participants according to MetS status. Mean ± SD, tested after log transformation of the data; tested by the one-way analysis of variance with the *Bonferroni* method and with the general linear model after adjustment for confounding factors (age, total calorie intake, cigarette smoking, alcohol drinking, and/or menopausal status); *P*_0_: Unadjusted *p*-value; *P*_1_: *P*-value adjusted for confounding factors. Values sharing the same alphabet indicate no statistically significant differences among them. GLP-1: Glucagon-like peptide-1; HOMA-IR: Homeostatic model assessment of insulin resistance; MetS: Metabolic syndrome.

The circulating GLP-1 levels and HOMA-IR scores in the total number of study participants were compared according to MetS status (Figure 1). The circulating GLP-1 levels and HOMA-IR scores were significantly higher for those in the MetS group than those in the Super-healthy and MetS risk carriers groups. These patterns remained after the data had been adjusted for age, TCI, cigarette smoking, alcohol drinking, and menopausal status.

### 3.3. Relationship among GLP-1 or HOMA-IR, MetS Risk Factors, and Inflammation in the Women According to Menopausal Status

After an adjustment for confounding factors, the circulating GLP-1 levels were found to be significantly positively correlated with the HOMA-IR scores in the total group of participants (*n* = 140, r = 0.274, *p* = 0.004) and in the premenopausal group (*n* = 75, r = 0.364, *p* = 0.005), but not in the postmenopausal group (*n* = 65, r = 0.214, *p* = 0.154). The circulating GLP-1 levels correlated significantly with the WC, BP, and hs-CRP measurements in the total group after the adjustment. These patterns were particularly obvious in the postmenopausal women than in the premenopausal participants (Table 2). The HOMA-IR scores correlated significantly with all of the parameters (except hs-CRP) in the total group of women. However, the significance for WC, systolic blood pressure (SBP), HbA1c, and HDL-C disappeared in the premenopausal group, as did the significance for BP, HbA1c, and HDL-C in the postmenopausal group (Table 2).

### 3.4. Circulating GLP-1 Levels and HOMA-IR Scores According to the Number of MetS Risk Factors

**Figure 2:**
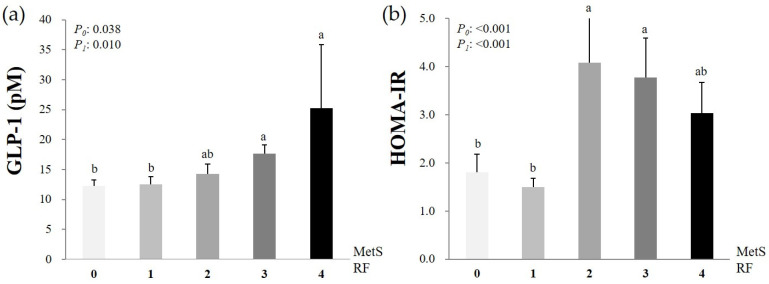
Circulating GLP-1 levels (**a**) and HOMA-IR scores (**b**) according to the number of MetS RFs. Mean ± SD, tested after log transformation of the data; tested by the one-way analysis of variance with the *Bonferroni* method and by the general linear model after adjustment for confounding factors (age, total calorie intake, cigarette smoking, alcohol drinking, and/or menopausal status); *P*_0_: Unadjusted *p*-value, *P*_1_: *P*-value adjusted for confounding factors. Values sharing the same alphabet indicate no statistically significant differences among them. GLP-1: Glucagon-like peptide-1; HOMA-IR: Homeostatic model assessment of insulin resistance; MetS RFs: Metabolic syndrome risk factors. Zero MetS RFs, *n* = 61; 1 MetS RF, *n* = 33; 2 MetS RFs, *n* = 27; 3 MetS RFs, *n* = 16; 4 MetS RFs, *n* = 3.

Figure 2 shows the circulating GLP-1 levels and HOMA-IR scores according to the number of MetS RFs. Generally, the circulating GLP-1 levels increased proportionally to the number of MetS RFs. By contrast, the HOMA-IR scores were higher in the participants with two or three MetS RFs than in those with zero or only one MetS RF.

### 3.5. Circulating GLP-1 Levels and HOMA-IR Scores According to Obesity and MetS Status

**Figure 3:**
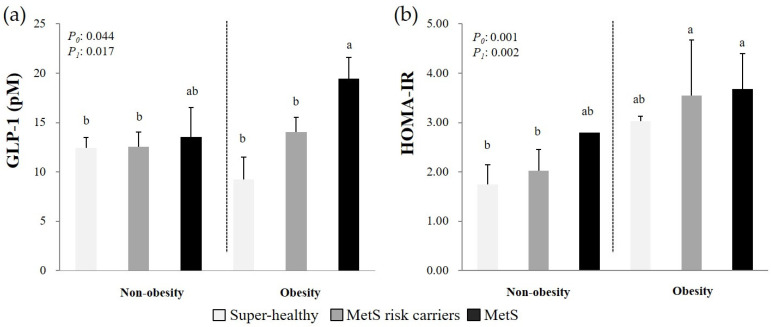
Circulating GLP-1 levels (**a**) and HOMA-IR scores (**b**) according to the combination of obesity and MetS status. Mean ± SD, tested after log transformation of the data; tested by the one-way analysis of variance with the *Bonferroni* method and with the general linear model with LSD after adjustment for confounding factors (age, total calorie intake, cigarette smoking, alcohol drinking, and menopausal status); *P*_0_: Unadjusted *p*-value, *P*_1_: *P*-value adjusted for confounding factors. Values sharing the same alphabet indicate no statistically significant differences among them. GLP-1: Glucagon-like peptide-1; HOMA-IR: Homeostatic model assessment of insulin resistance; MetS: Metabolic syndrome. Non-obesity group: Super-healthy, *n* = 57; MetS risk carriers, *n* = 31; and MetS, *n* = 2. Obesity group: Super-healthy, *n* = 4; MetS risk carriers, *n* = 29; and MetS, *n* = 17.

Figure 3 presents the circulating GLP-1 levels and HOMA-IR scores according to obesity and MetS status. The circulating GLP-1 levels were significantly higher in the women in the MetS group who were obese than in all the individuals in the Super-healthy and MetS risk carriers groups (*P*_0_ = 0.044, *P*_1_ = 0.017). By contrast, the HOMA-IR scores were higher in the individuals with an obesity status and at least one MetS RF than in the women without MetS RFs or with one or two MetS RFs and who were not obese.

## 4. Discussion

This study was performed to investigate whether circulating GLP-1 levels could be used as an early indicator of MetS risk in Korean women without a diagnosed disease. The fasting plasma GLP-1 levels were significantly higher in the MetS group than in subjects from the Super-healthy and MetS risk carriers groups. The increase in circulating GLP-1 levels was also proportional to the increase in the number of MetS RFs and in particular, was significantly correlated with the WC and BP measurements. These patterns were more distinct in the postmenopausal, than in the premenopausal women. On the basis of these results, we concluded that the circulating GLP-1 level can very likely be used as a potential early indicator of MetS risk in women without a diagnosed disease.

Given that the global prevalence of overweight and obesity is expected to reach 57.8% by 2030 [5], the burden of metabolic abnormalities and the risks of chronic degenerative diseases such as CVD, diabetes, and cancer are likely to increase, as well. According to a recent report in Korea [4], the incidence of obesity, especially abdominal obesity, is continuously increasing with the rising prevalence of related metabolic diseases (i.e., diabetes, hypertension, and dyslipidemia). Therefore, many clinicians and scientists have been searching for ways to prevent and manage the risk of MetS. As mentioned in the Introduction, the effectiveness of GLP-1 treatment in controlling blood glucose levels, appetite, and energy consumption has been proven in recent studies [10,11,14,15,16,35]. GLP-1 stimulates the signaling pathways downstream of cyclic adenosine monophosphate (cAMP) and protein kinase A (PKA) in the nucleus tractus solitarii, resulting in the suppression of food ingestion [36]. Specifically, GLP-1R activation in the hindbrain suppresses energy ingestion via the PKA-mediated reduction of adenosine monophosphate protein kinase activity and the concurrent upregulation of the components of the mitogen-activated protein kinase/extracellular signal-related kinase-1/2 pathways [36].

The weight loss effect of GLP-1 is induced through the activation of invariant natural killer T (iNKT) cells and the triggering of fibroblast grow factor 21 (FGF21) [35]. This GLP-1-induced activation of the iNKT cells results in a strong weight loss and sugar control in obesity and is associated with the thermogenic browning of white fat [35]. Moreover, the FGF21 molecules secreted by iNKT cells play an important role in activating the thermogenic process in brown adipose tissue [37]. Inhibitors of DPP-4 (the enzyme that deactivates GLP-1) and GLP-1 analogs that have functions similar to those of GLP-1 have been commercialized for the treatment of diabetes [12,38] and have also recently been used for the treatment of obesity [12,38,39,40]. In addition to the GLP-1R in the pancreas, GLP-1 can bind to its receptor in various other organs (e.g., brain, stomach, muscles, liver, and heart) to perform diverse functions [36]. For example, GLP-1 participates in neuroprotection, the signaling of appetite suppression by the brain, the postponement of gastric emptying, the decrease of gastric acid output and gastric motility in the stomach, the increasing of glucose uptake in muscle, the inhibition of hepatic IR, the alleviation of hepatic steatosis in the liver, positive inotropic and chronotropic effects, and the increase of reperfusion after cardiac ischemia [36,41]. Thus, the increase in circulating GLP-1 levels in response to any metabolic abnormality can be expected to have a positive effect in preventing chronic degenerative diseases by regulating the metabolic processes occurring in various organs. Additionally, such circulating GLP-1 levels would have the potential to be used as a predictive marker for the early diagnosis of MetS risk. The findings of this study showed that circulating GLP-1 levels were significantly higher in women in the MetS group than in those in the Super-healthy and MetS risk carriers groups, even after adjustment for age, TCI, smoking, drinking habits, and menopausal status. In particular, the circulating GLP-1 concentration was highly correlated with obesity (as estimated by BMI and WC), BP, and HOMA-IR, as well as with hs-CRP, an inflammatory marker. When the participants were grouped according to the risk level of each MetS RF, the circulating GLP-1 concentration was also notably higher in the individuals with high WC and high BP measurements (data not shown). A reason for this phenomenon is that the central fat distribution acts as a major factor in the pathogenesis of metabolic abnormalities [42]. It has been reported that GLP-1 enhances sodium excretion and reduces both H^+^ secretion and glomerular hyperfiltration [41,43,44,45]. When GLP-1 binds to its receptors in the kidneys, the cAMP/PKA signaling pathway is activated, leading to the phosphorylation of the PKA connection position at the COOH end of Na(+)/H(+) exchanger isoform 3 (NHE3) [44]. GLP-1 also has diuretic and natriuretic effects that are mediated by changes in the renal hemodynamics and by the downregulation of NHE3 activity in the renal proximal tubule [45]. Additionally, GLP-1 prevents hyperglycemia-related inflammation and apoptosis by lowering the blood sugar levels in the brain via the downregulation of PKA and phosphoinositide 3-kinase activities [36]. In this study, the hs-CRP concentrations correlated positively with the circulating GLP-1 levels, indicating that an increased GLP-1 level in circulation is a reward mechanism for the maintenance of homeostasis. Our results showed that the circulating GLP-1 levels were higher in the participants with MetS RFs and were proportional to the number of MetS RFs. By contrast, the GLP-1 concentration was not associated with the blood levels of fasting glucose, TG, and HDL-C in a statistically significant manner. This was likely due to the fact that the study participants were all relatively healthy individuals who had no diagnosed chronic diseases such as diabetes or CVD, and thus, their glucose or lipid metabolism-associated homeostasis was well maintained.

Whereas most previous studies were focused on GLP-1 as a therapeutic agent for the treatment of diabetes and obesity [11,12,13,14,15,16,30,38], we measured the circulating GLP-1 levels in the body under the fasting state. As mentioned above, GLP-1 plays an important role in regulating glucose homeostasis [11,12,13]. Fasting levels of plasma GLP-1 were significantly higher in patients with T2D than people with NGT, but postprandial plasma GLP-1 levels and area under curve were significantly lower in patients with T2D than people with NGT [11,14]. Therefore, the high levels of circulating GLP-1 in our study participants may be a compensation for maintaining homeostasis in response to metabolic changes. When the women were further grouped according to menopausal status, the relationships between the circulating GLP-1 levels and metabolic parameters were more distinct in the postmenopausal group, with female hormones being the likely cause [46,47]. It was well reported that estradiol has anti-inflammatory and antioxidative effects, thereby indirectly enhancing the insulin receptor function [46,47]. This hormone also has a direct effect on glucose uptake by the skeletal muscle and adipocytes, leading to the lowering of the blood glucose concentration [46,48]. In women, the estrogen hormones regulate fat accumulation in the subcutaneous tissue, especially in the femoral and gluteal regions [49]. However, abdominal fat accumulation ensues when estrogen levels are decreased or depleted, as seen in the case of postmenopausal women [47,49].

In this study, the positive correlation between increasing circulating GLP-1 concentrations and the number of MetS RFs was more marked in the individuals from the MetS group with an obesity status than in all participants from the Super-healthy and MetS risk carriers groups. By contrast, the HOMA-IR scores appeared to be more strongly associated with the obesity status than with metabolic abnormalities.

Taken together, these results indicate that the circulating GLP-1 levels consistently and sensitively reflect the risk of MetS in Korean women without a diagnosed disease. This was more distinct in women who are postmenopausal, suggesting that GLP-1 may indeed be a useful early indicator of MetS risk. However, this study had several limitations. First, since it focused only on fasting blood GLP-1 concentrations and metabolic parameters, further analyses of the association of the metabolic parameters with circulating GLP-1 concentrations in both the fasting and postprandial states are required. Second, an increase in the number of study participants and the inclusion of men into the cohort are needed to confirm the validity of the present study’s results.

## 5. Conclusions

Despite the small number of participants in this study, a notable relationship between the circulating GLP-1 levels and MetS RFs was observed. The results suggest that the blood GLP-1 concentration may be a potential early indicator of MetS risk in women without a diagnosed disease, especially in those who are postmenopausal, and could serve as a helpful strategy for preventing the development of chronic degenerative diseases.

## Figures and Tables

**Table 1:** Baseline characteristics and biochemical parameters of the study participants according to the metabolic syndrome (MetS) status.

	Super-Healthy (*n* = 61)			MetS Risk Carriers (*n* = 60)			MetS (*n* = 19)			*p*-Value
Age (years)	43.3	±	1.53 ^b^	46.8	±	1.77 ^b^	58.4	±	2.54 ^a^	<0.001
Body mass index (kg/m^2^)	22.1	±	0.24 ^c^	25.0	±	0.42 ^b^	26.5	±	0.66 ^a^	<0.001
Systolic BP (mmHg)	110.7	±	1.11 ^c^	118.2	±	1.88 ^b^	130.8	±	2.21 ^a^	<0.001
Diastolic BP (mmHg)	70.6	±	0.70 ^b^	76.5	±	1.27 ^a^	80.5	±	2.16 ^a^	<0.001
Waist circumference (cm)	75.0	±	0.67 ^b^	85.2	±	1.24 ^a^	88.6	±	1.69 ^a^	<0.001
Total calorie intake (kcal/day)	1753.1	±	64.5	1726.2	±	53.6	1550.1	±	99.7	0.237
Carbohydrates (% of TCI)	57.6	±	1.26	57.9	±	1.25	61.6	±	2.58	0.302
Fat (% of TCI)	25.5	±	1.08	24.4	±	0.95	20.7	±	1.99	0.076
Protein (% of TCI)	16.8	±	0.44	17.7	±	0.62	17.7	±	0.97	0.476
Obesity (BMI ≥ 25 kg/m^2^) (%)	6.6			48.3			89.5			<0.001
Current cigarette smoker (%)	1.7			8.3			0.0			0.121
Current alcohol drinker (%)	68.3			56.7			42.1			0.104
Menopause (%)	32.8			46.7			89.5			<0.001
Glucose (mg/dL) ^§^	83.7	±	1.04 ^c^	92.6	±	2.44 ^b^	109.3	±	3.74 ^a^	<0.001
HbA1c (%) ^§^	5.26	±	0.05 ^c^	5.55	±	0.07 ^b^	5.91	±	0.16 ^a^	<0.001
Insulin (μIU/mL)	8.39	±	1.54	10.6	±	1.62	13.6	±	2.23	0.260
C-peptide (ng/mL) ^§^	1.90	±	0.24 ^b^	2.26	±	0.23 ^ab^	2.91	±	0.40 ^a^	0.035
Triglyceride (mg/dL)	66.8	±	3.40 ^c^	99.5	±	6.00 ^b^	170.6	±	16.4 ^a^	<0.001
HDL-cholesterol (mg/dL)	67.8	±	1.42 ^a^	59.8	±	2.01 ^b^	52.2	±	2.41 ^c^	<0.001
LDL-cholesterol (mg/dL) ^§^	115.7	±	3.43 ^b^	124.2	±	4.32 ^ab^	135.9	±	8.38 ^a^	0.078
Total cholesterol (mg/dL) ^§^	187.8	±	3.37	196.1	±	4.53	205.7	±	9.51	0.157
hs-CRP (mg/dL) ^§^	0.50	±	0.10 ^b^	1.52	±	0.66 ^a^	0.87	±	0.21 ^a^	0.009
FSH (mIU/mL) ^§^	27.3	±	3.99 ^b^	31.7	±	4.20 ^b^	56.7	±	5.88 ^a^	0.001
Estradiol (pg/mL) ^§^	87.2	±	13.9 ^a^	101.0	±	17.8 ^a^	27.2	±	13.7 ^b^	0.002

Mean ± SD or %, ^§^ tested after long-transformed, tested by the one-way analysis of variance with the *Bonferroni* method or Chi-square test. Values sharing the same alphabet indicate no statistically significant differences among them. BP: Blood pressure; BMI: Body mass index; FSH: Follicle-stimulating hormone; HbA1c: Hemoglobin A1c; HDL: High-density lipoprotein; hs-CRP: High-sensitivity C-reactive protein; LDL: Low-density lipoprotein; MetS: Metabolic syndrome; TCI: Total calorie intake.

**Table 2:** Relationships among GLP-1 or HOMA-IR, MetS RFs, and inflammation in the women according to the menopausal status.

Variables	Status	WC		SBP		DBP		FG ^§^		HbA1c ^§^		TG ^§^		HDL-C		hs-CRP ^§^		
GLP-1 ^§^	Total	0.295	**	0.322	**	0.237	**	−0.006		−0.015		0.102		−0.014		0.281	**
	Pre	0.165		0.298	*	0.193		−0.043		−0.098		0.088		−0.061		0.230	^ø^
	Post	0.461	**	0.354	**	0.340	**	0.011		0.100		0.203		0.019		0.276	*
HOMA-IR ^§^	Total	0.332	**	0.212	*	0.226	*	0.580	**	0.234	*	0.326	**	−0.212	*	0.016	
	Pre	0.181		*0.225*	^ø^	0.340	**	0.474	**	*0.227*	^ø^	0.318	*	−0.156		0.091	
	Post	0.542	**	0.207		0.150		0.661	**	*0.248*	^ø^	0.338	*	−0.243		0.100	

Correlation coefficient, ^§^ tested after log transformation of the data; tested by the partial correlation analysis after adjustment for age, total calorie intake, cigarette smoking, alcohol drinking, and/or menopausal status; ^ø^ *p* < 0.1, * *p* < 0.05, ** *p* < 0.01. Total: Total group of women (*n* = 140), Pre: Premenopausal group (*n* = 75), Post: Postmenopausal group (*n* = 65); DBP: Diastolic blood pressure; FG: Fasting glucose; GLP-1: Glucagon-like peptide-1; HbA1c: Hemoglobin A1c; HDL-C: High-density lipoprotein cholesterol; hs-CRP: High-sensitivity C-reactive protein; HOMA-IR: Homeostatic model assessment of insulin resistance; SBP: Systolic blood pressure; TG: Triglyceride; WC: Waist circumference.

## Data Availability

The data presented in this study are not publicly available, but are available from the corresponding author on reasonable request.
